# SPI1 Mediates N-Myristoyltransferase 1 to Advance Gastric Cancer Progression via PI3K/AKT/mTOR Pathway

**DOI:** 10.1155/2023/2021515

**Published:** 2023-03-17

**Authors:** Ping Qiu, Xing Li, Min Gong, Ping Wen, Jianbo Wen, Linfang Xu, Guiliang Wang

**Affiliations:** Department of Gastroenterology, Jiangxi Pingxiang People's Hospital, Pingxiang, Jiangxi, China

## Abstract

Gastric cancer (GC) is a common digestive tract malignancy worldwide. N-myristoyltransferase 1 (NMT1) has been implicated in many cancers, but its association with gastric cancer remains to be clarified. Thus, this paper elucidated the role of NMT1 in GC. The NMT1 expression level in GC and normal tissue samples as well as the relationship between NMT1 high or low expression and overall survival in GC was analyzed via GEPIA. GC cells were transfected with NMT1 or SPI1 overexpression plasmid and short hairpin RNA against NMT1 (shNMT1) or shSPI1. NMT1, SPI1, p-PI3K, PI3K, p-AKT, AKT, p-mTOR, and mTOR levels were detected through qRT-PCR and western blot. MTT, wound healing, and transwell assays were applied to test cell viability, migration, and invasion. The binding relationship of SPI1 and NMT1 was determined through a dual-luciferase reporter assay and chromatin immunoprecipitation. NMT1 was upregulated in GC, the high level of which connected with a poor prognosis. Overexpressed NMT1 elevated viability, migration rate, and invasion rate of GC cells, whereas NMT1 knockdown leads to the opposite results. Besides, SPI1 could bind to NMT1. Overexpressed NMT1 reversed the effects of shSPI1 on decreasing viability, migration, invasion, p-PI3K/PI3K, p-AKT/AKT, and p-mTOR/mTOR in GC cells, and NMT1 knockdown reversed the effects of SPI1 overexpression on increasing viability, migration, invasion, p-PI3K/PI3K, p-AKT/AKT, and p-mTOR/mTOR. SPI1 upregulated NMT1 to facilitate the malignant behaviors of GC cells through the PI3K/AKT/mTOR pathway.

## 1. Introduction

According to the statistics, China had over 679,000 new gastric cancer (GC) cases and 498,000 deaths caused by the disease in 2015, whose incidence and fatality have still been elevated in recent years, enabling GC to be one of the most diagnosed malignant tumors and the 2^nd^ leading cause of cancer-associated death in China [1]. *Helicobacter pylori* infection, age, family history, smoking, drinking, and diet were the risk factors for GC [2–4]. What is worse, GC lacks obvious symptoms at the early stage, and most of the patients with GC have already developed into the middle or late stage at the time of diagnosis, leading to a short survival time, poor prognosis, and high morbidity [5, 6]. At present, the primary treatment method for GC is surgery with chemotherapy and radiotherapy serving as adjuvant therapy. However, the results remain unsatisfactory due to the limited efficacy performed in advanced GC [6–8]. Increasing studies have been conducted on the molecular mechanism of cancer; for example, it has been reported that the TNF-*α*-308 G/A polymorphism may contribute to susceptibility to GC [9]. As the understanding of molecular mechanisms implicated in multiple cancers deepens, an emerging molecular targeted therapy attracts more and more attention, in which molecules connecting with cell growth, apoptosis, metastasis, invasion, and angiogenesis are regarded as a promising treatment approach for cancers because molecularly targeted agents can affect the biological behavior of tumor cells through regulating molecules intimately related to tumor onset and development, with the therapeutic effectiveness of that therapy also validated in GC [10, 11]. Hence, the identification of innovative biomarkers for GC diagnosis and prognosis may contribute to the early detection and treatment of GC.

N-myristoyltransferase 1 (NMT1) is an enzyme catalyzing the co/posttranslational myristoylation of more than 100 proteins coded by human genes, through which myristate is transferred to the N-terminal glycine of a series of substrate proteins [12–14]. Previous research studies have demonstrated that NMT1 plays an important part in a great number of diseases, including malignancies, which is mainly attributed to the participation of its substrates in cell transformation, signal cascade, and tumorigenesis [14, 15]. It has been reported that the suppression of NMT1 abolishes the function of Src on facilitating prostate cancer advancement, and NMT1 knockdown restrained breast cancer progression via JNK pathway triggered by stress [16, 17]. In addition, the use of NMT inhibitors brings about endoplasmic reticulum (ER) stress, cell cycle arrest, and apoptosis in HeLa cells, with the similar results determined in breast cancer and colon cancer [18]. Nevertheless, few works have expounded the effects of NMT1 on GC.

In this paper, we probed into the role and mechanisms of NMT1 in modulating GC cell viability, migration, and invasion, intending to investigate the feasibility of NMT1 as a target for GC treatment.

## 2. Materials and Methods

### 2.1. Bioinformatics

The differential expressed NMT1 of GC (*n* = 408) and normal tissues (*n* = 211) as well as the relationship between NMT1 high (*n* = 605) or low (*n* = 270) expression and overall survival in GC was analyzed by gene expression profiling interactive analysis (GEPIA; http://gepia.cancer-pku.cn/). The binding sites between NMT1 and SPI1 were analyzed by JASPAR (https://jaspar.genereg.net/).

### 2.2. Cell Culture

Human normal gastric mucosal cell line GES-1 and GC cell line AGS, HGC27, SNU-5, and MKN-45 were bought from Procell (CL-0563, CL-0022, CL-0107, CL-0444, and CL-0292, Wuhan, China). GES-1 and MKN-45 cells were cultured in RPMI-1640 medium (PM150110, Procell, China) enriched with 10% fetal bovine serum (FBS; 164210, Procell, China) and 1% penicillin-streptomycin solution (P/S; PB180120, Procell, China). AGS cells were incubated in Ham's F-12 Nutrient Mixture (PM150810, Procell, China) with 10% FBS and 1% P/S; HGC27 cells were cultured in RPMI-1640 medium enriched with 20% FBS and 1% P/S; SNU-5 cells were incubated in Iscove's modified Dulbecco medium (IMDM; PM150510, Procell, China) containing 20% FBS and 1% P/S. All cells were cultured at 37°C with 5% CO_2_.

### 2.3. Cell Transfection

NMT1 or SPI1 overexpression plasmid based on pcDNA3.1/+vector (V79020, Thermo Fisher, Waltham, MA, USA) and the empty vector that served as a negative control (NC), short hairpin RNA against NMT1 (shNMT1, GAGCCAAAAAGAAGAAAAAGAAA) or SPI1 (shSPI1, GCCCTATGACACGGATCTATACC), and their negative control (shNC) that ordered from Genepharma (China) were transfected into AGS and SNU-5 cells using Lipofectamine 2000 (11668500, Thermo Fisher, USA). In short, cells (1 × 10^5^/well) that were seeded in 24-well plates were incubated until 80% confluence. Subsequently, NMT1/SPI1 overexpression plasmid (0.8 *μ*g) or shNMT1/shSPI1 (20 pmol) was diluted in IMDM (50 *μ*L), and another 50 *μ*L of IMDM was applied to dilute 2.0 *μ*L (for NMT1/SPI1 overexpression plasmid) or 1 *μ*L (for shNMT1/shSPI1) Lipofectamine 2000 for 5 min at room temperature. After a mixture of diluted NMT1/SPI1 overexpression plasmid or shNMT1/shSPI1 and the respective diluted Lipofectamine 2000 at room temperature for 20 min, complexes (100 *μ*L per well) were added, followed by cell culture at 37°C for 24 or 48 h [19].

### 2.4. Dual-Luciferase Reporter Assay

HEK293 cells (CL-0001, Procell Life, China) were incubated in minimum essential medium (MEM; PM150467, Procell, China) containing 1% P/S and 10% FBS. A reporter vector (E1330, Promega Corporation, Madison, WI, USA) with human NMT1 3′-untranslational region (UTR) sequences was constructed to obtain wild-type NMT1 (NMT1-WT; AAAACAGAGGAAATAACACG), whereas the other reporter vector with mutative NMT1 (NMT1-MUT; CCCCCCACAACCCTCCCCCG) 3′-UTR sequences in the SPI1 promoter region was considered as the NC. NMT1-WT or NMT1-MUT and SPI1 overexpression plasmid were transfected into HEK293 cells for 24 h. The luciferase activity was accessed by a dual-luciferase reporter assay system (E1910, Promega, USA) [20].

### 2.5. Chromatin Immunoprecipitation (ChIP)

ChIP assay was performed with the ChIP kit (P2078, Beyotime, China) according to the producer's directions [21]. AGS cells seeded in 10 cm dishes (1 × 10^6^ cells) were crosslinked with 1% methanal and then incubated for 10 min. Subsequently, 125 mM of glycine solution was added and stood at 25°C for 5 min. Following solution removal with PBS, the cells were lysed by SDS lysis buffer on ice. Sonication was performed to shear genomic DNA with a sonicator (S4000, Misonix, USA) at 4°C so that most of the DNA was fragmented to 400–800 bp in size. After the centrifugation (12,000 g, 4°C, 5 min), the supernatants (0.2 mL) were diluted with 1.8 mL of ChIP dilution buffer. The SPI1 (ab227835, Abcam, UK) and IgG (ab171870, Abcam, UK) antibodies were added and incubated at 4°C overnight. Thereafter, 60 *μ*L of protein A + G Agarose/Salmon Sperm DNA was added and mixed for 60 min at 4°C to precipitate proteins recognized by the primary antibodies. Next, 250 *μ*L of elution buffer was added to elute DNA, followed by incubation with 4.8 µL of NaCl (5 M) and 2 *μ*L of RNase A (10 mg/mL) at 65°C overnight. After the samples were purified with phenol/chloroform, the NMT1 expression level was examined by quantitative real-time PCR (qRT-PCR).

### 2.6. MTT Assay

MTT assay was applied in the detection of cell viability through a MTT kit (abs50010, Absin, China) [22]. AGS or SNU-5 cells (5 × 10^3^ per well) were added into each well of 96-well plates and cultured for 24, 48, or 72 h. Later, 10 *μ*L/well MTT solution was supplemented into each well. After the 4 h culture at 37°C, 100 *μ*L formazan dissolving solution was added for another 4 h incubation, and the absorbance was tested at 450 nm via the microplate reader (SpectraMax 190, Molecular Devices, China).

### 2.7. Wound Healing Assay

AGS or SNU-5 cells were seeded in a 6-well plate at a density of 5 × 10^5^ cells per well and cultured overnight until the cell confluence reached about 80%. Next, two parallel lines spaced at a distance of 1 cm were scratched with the tip of the pipette. After discarding of cell debris with PBS, cells were cultured at 37°C for 48 h and viewed under the microscope (magnification: ×100) with images recorded [23].

### 2.8. Transwell Assay

Transwell chamber (8 *μ*m pore size; Corning, USA) precoated with Matrigel (356234, Corning, USA) was applied to detect the cell invasion [17]. Simply put, AGS or SNU-5 cells (1 × 10^5^ cells/well) were suspended in the upper chamber with 200 mL serum-free medium, and 500 mL medium with 10% FBS was added into the lower chamber as an attractant. After 48 h culture, the invading cells in the lower chamber were fixed with paraformaldehyde (BL-G002, SenBeiJia, Nanjing, China) and stained with 0.1% crystal violet (BP-DL134, SenBeiJia, China). The number of invaded cells was counted with the microscope (×250).

### 2.9. qRT-PCR

Total RNA in cells was extracted by Trizol (KGA1202, KeyGEN Biotech, China), and then the synthesis of cDNA was operated through reverse transcription by the BeyoRT III cDNA Synthesis Kit (D7178, Beyotime, China). Next, cDNA and primers for detecting mRNA, as well as SYBR Green Supermix (A46113, Applied Biosystems, USA) for labeling, were added to a Fast7500 real-time PCR system (ABI, USA) to conduct the qPCR, and the thermal cycling program included predenaturation at 95°C for 2 min, 40 cycles of 95°C for 5 s and 60°C for 30 s. Following these, the internal reference gene GAPDH was utilized to calculate the mRNA level of the genes listed in Table 1 according to the 2^−ΔΔCt^ formula [24].

### 2.10. Western Blot

Western blot was performed as described previously [17]. RIPA buffer (R0278, Sigma–Aldrich, USA) was applied to lyse and extract the total protein from cells. After the protein concentration was measured by BCA protein quantitation kit (55R-1544, Fitzgerald, USA), sodium dodecyl sulfate-polyacrylamide gel electrophoresis (SDS-PAGE) gels were exploited to separate the protein (45 *μ*g) and marker (5 *μ*L; G2086, Servicebio, China), which was then moved to polyvinylidene fluoride membranes (24937, Sigma-Aldrich, China). The membranes were sealed by defatted milk and subsequently incubated with primary antibodies (Table 2) at 4°C overnight. Next, the membranes were incubated with the goat anti-rabbit (ab97051, 1 : 5000, Abcam) or rabbit-anti-mouse secondary antibody (ab6709, 1 : 2000, Abcam) for 2 h. An excellent chemiluminescent substrate detection kit (E-BC-R347, Elabscience, China) was used to measure the protein bands, and an eZwest Lite auto western blotting system (Genscript, Piscataway, NJ, USA) was employed to scan the bands.

### 2.11. Statistical Analysis

GraphPad 8.0 (GraphPad Software, USA) was adopted to analyze statistics. All results were expressed as mean ± standard deviation from at least triplicate experiments; paired sample *t * test was used to compare the paired data between the two groups while independent samples *t* test was utilized to compare the two groups of independent sample data. A one-way ANOVA was used to evaluate the significance among multiple groups followed by the Bonferroni post hoc test. *p* < 0.05 implicated a statistically significant difference.

## 3. Results

### 3.1. NMT1 Was Upregulated in GC Tissues and Intimately Associated with the Prognosis of GC Patients

**Figure 1:**
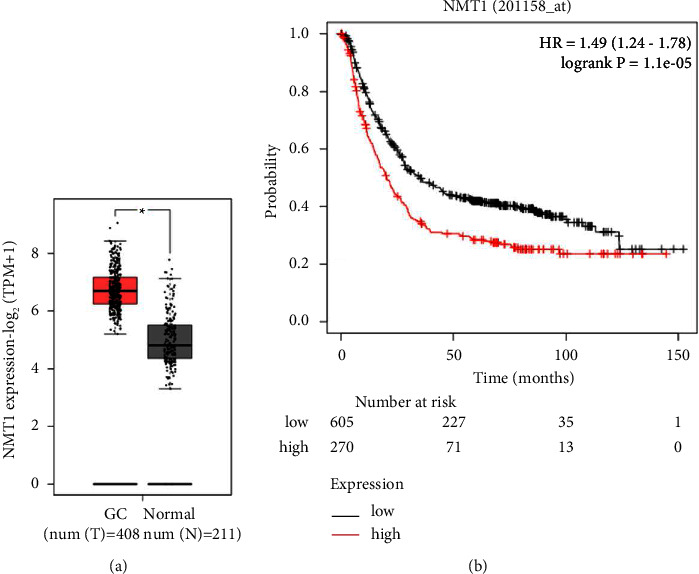
NMT1 was upregulated in GC tissues and might be a potential biomarker of GC diagnosis. (a) The differentially expressed NMT1 between GC (*n* = 408) and normal (*n* = 211) tissue samples was analyzed by GEPIA (http://gepia.cancer-pku.cn/). (b) The overall survival of GC patients with high (*n* = 605) or low (*n* = 270) NMT1 expression was analyzed by GEPIA, *p* = 1.1e-05. ^∗^*p* < 0.05 vs. Normal tissues. NMT1, N-myristoyltransferase 1; GC, gastric cancer; GEPIA, gene expression profiling interactive analysis.

GEPIA analysis exhibited that NMT1 was highly expressed in GC tissue samples relative to normal tissue samples (Figure 1(a); *p* < 0.05). In addition, the survival time of GC patients with NMT1 high expression was shorter than those with NMT1 low expression (Figure 1(e); *p* = 1.1*e* − 05). Taken all above together, it was suggested that NMT1 was markedly upregulated in human GC tissues, and the elevated level of NMT1 was related to a poor prognosis.

### 3.2. NMT1 Was Upregulated in GC Cells and Modulated GC Cell Viability, Migration, and Invasion

**Figure 2:**
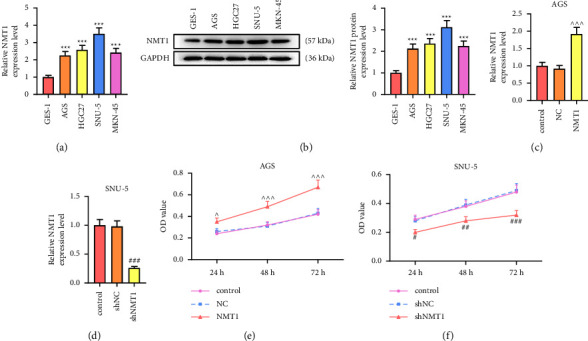
NMT1 was upregulated in GC cells and modulated GC cell viability. (a) NMT1 mRNA expression between human GC cell lines (AGS, HGC27, SNU-5, and MKN-45) and normal cell line GES-1 was detected through qRT-PCR. GAPDH was the loading control. (b) NMT1 protein expression between human GC cell lines (AGS, HGC27, SNU-5, and MKN-45) and normal cell line GES-1 was tested by western blot. GAPDH was the loading control. (c) NMT1 mRNA expression of AGS cells was detected through qRT-PCR after transfection of NMT1 overexpression plasmid. GAPDH was the loading control. (d) NMT1 mRNA expression of SNU-5 cells was detected through qRT-PCR after transfection of shNMT1. GAPDH was the loading control. (e) OD value of AGS cells at 24, 48, or 72 h was assessed by MTT assay after transfection of NMT1 overexpression plasmid. (f) OD value of SNU-5 cells at 24, 48, or 72 h was assessed by MTT assay after transfection of shNMT1. ^∗∗∗^*p* < 0.001 vs. GES-1 cells;^∧^*p* < 0.05, ^∧∧∧^*p* < 0.001 vs. NC group; ^#^*p* < 0.05, ^##^*p* < 0.01, ^###^*p* < 0.001 vs. shNC group. All experiments were repeated independently at least three times. Data were performed as the means ± standard deviation. NMT1, N-myristoyltransferase 1; GC, gastric cancer; qRT-PCR, quantitative reverse transcription-polymerase chain reaction; GAPDH, glyceraldehyde-3-phosphate dehydrogenase; shNMT1, short hairpin RNA against NMT1; OD, optical density; NC, negative control.

**Figure 3:**
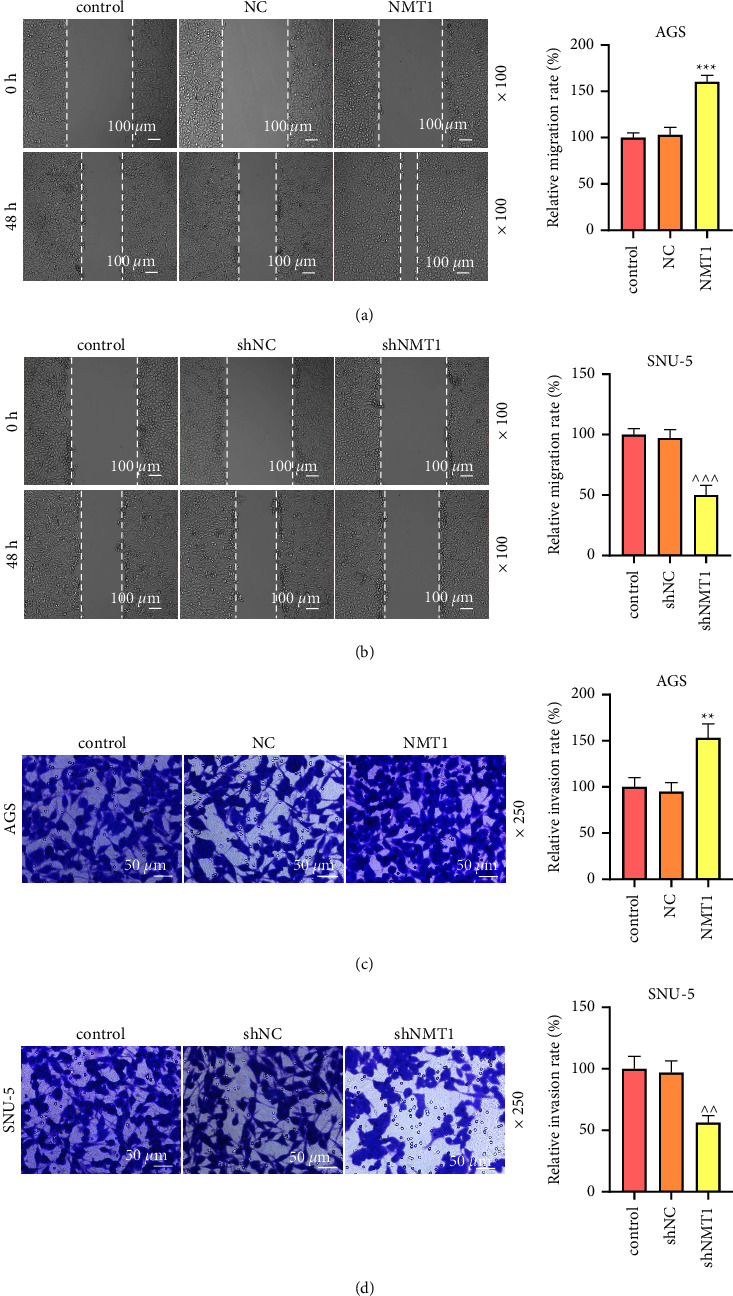
NMT1 modulated GC cell migration and invasion. (a) Migration rate of AGS cells at 48 h was evaluated through wound healing assay after transfection of NMT1 overexpression plasmid. (b) Migration rate of SNU-5 cells at 48 h was evaluated through wound healing assay after transfection of shNMT1. (c) Invasion rate of AGS cells at 48 h was determined by transwell assay after transfection of NMT1 overexpression plasmid. (d) Invasion rate of SNU-5 cells at 48 h was determined by Transwell assay after transfection of shNMT1. ^∗∗^*p* < 0.01, ^∗∗∗^*p* < 0.001 vs. NC group; ^∧∧^*p* < 0.01, ^∧∧∧^*p* < 0.001 vs. shNC group. All experiments were repeated independently at least three times. Data were performed as the means ± standard deviation. NMT1, N-myristoyltransferase 1; GC, gastric cancer; shNMT1, short hairpin RNA against NMT1; NC, negative control.

Similar to the results of NMT1 expression in GC tissues, NMT1 mRNA and protein levels in GC cell lines (AGS, HGC27, SNU-5, and MKN-45) were evidently increased in comparison with the normal cell line GES-1 (Figures 2(a) and 2(b); *p* < 0.001). In order to explore the role of NMT1 in GC cells, we established a NMT1 overexpression model in AGS cells that expressed relatively low expression of NMT1 as well as the NMT1 silence model in SNU-5 cells that expressed relatively high expression of NMT1. The results of qRT-PCR validated the successful establishment of models, which was evidenced by the fact that the NMT1 mRNA level was dramatically elevated in AGS cells transfected with an NMT1 overexpression plasmid while markedly decreased in SNU-5 cells transfected with shNMT1 in contrast with their respective negative controls (Figures 2(c) and 2(d); *p* < 0.001). During the MTT assay, the OD value at 24, 48, or 72 h of AGS cells in the NMT1 group was evidently higher than that in the NC group (Figure 2(e); *p* < 0.05), whereas that of SNU-5 cells in shNMT1 group was appreciably lower than that in the shNC group (Figure 2(f); *p* < 0.05). In addition, the NMT1 overexpression plasmid raised the migration and invasion rates of AGS cells, whereas shNMT1 declined the migration and invasion rates of SNU-5 cells (Figures 3(a)–3(d); *p* < 0.01). These data indicated that overexpressed NMT1 promoted, but its knockdown restrained GC cell viability, migration, and invasion.

### 3.3. SPI1 Could Bind to NMT1

**Figure 4:**
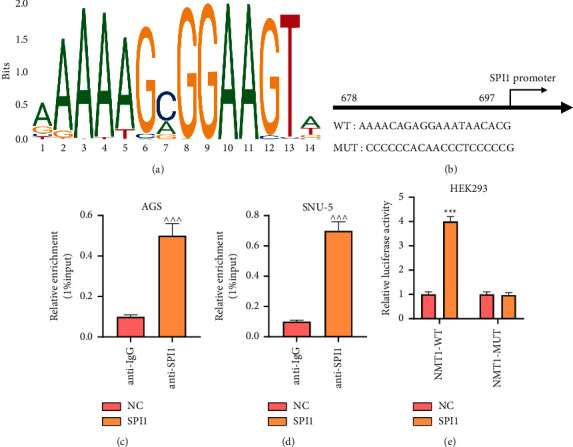
SPI1-targeted NMT1. (a, b) The binding sites between SPI1 and NMT1 were predicted by JASPAR database (https://jaspar.genereg.net/). (c, d) The enrichment of NMT1 in the anti-IgG and anti-SPI1 groups of AGS (c) and SNU-5 (d) cells was determined by chromatin immunoprecipitation assay. (e) Luciferase reporter assay was applied to determine the binding between SPI1 and NMT1. ^∗∗∗^*p* < 0.001 vs. NC group; ^∧∧∧^*p* < 0.001 vs. anti-IgG. All experiments were repeated independently at least three times. Data were performed as the means ± standard deviation. NMT1, N-myristoyltransferase 1; NMT1-WT, wild-type NMT1; NMT1-MUT, mutative NMT1; NC, negative control.

Based on the analysis of JASPAR, there are binding sites between NMT1 and SPI1 (Figures 4(a) and 4(b)). Next, the results of the ChIP assay evidenced that the enrichment of NMT1 was largely increased in the anti-SPI1 group (Figures 4(c) and 4(d); *p* < 0.001). Besides, the dual-luciferase reporter assay presented that the co-transfection of SPI1 overexpression plasmid and NMT1-WT reduced the luciferase activity of HEK293 cells in comparison with co-transfection of NC and NMT1-WT (Figure 4(e); *p* < 0.001), while no obvious difference was performed in the luciferase activity of the NMT1-MUT groups (Figure 4(e)). Therefore, the above data implicated that SPI1 could bind to NMT1.

SPI1 regulates NMT1 to mediate viability, migration, and invasion in GC cells through the PI3K/AKT/mTOR pathway.

**Figure 5:**
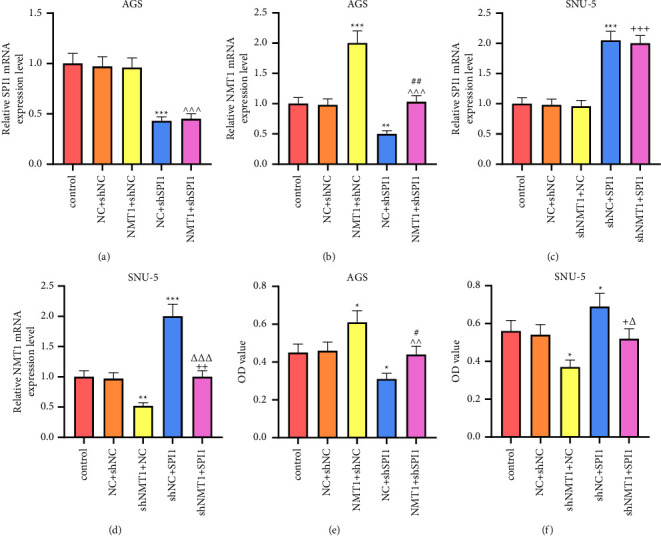
SPI1 regulated NMT1 to mediate GC cell viability. (a, b) SPI1 expression (a) and NMT1 mRNA expression (b) of AGS cells were detected through qRT-PCR after transfection of NMT1 overexpression plasmid and shSPI1. GAPDH was the loading control. (c, d) The mRNA expressions of SPI1 (c) and NMT1 (d) of SNU-5 cells were detected through qRT-PCR after transfection of shNMT1 and SPI1 overexpression plasmid. GAPDH was the loading control. (e) OD value of AGS cells at 48 h was assessed through MTT assay after transfection of NMT1 overexpression plasmid and shSPI1. (f) OD value of SNU-5 cells at 48 h was assessed through MTT assay after transfection of shNMT1 and SPI1 overexpression plasmid. ^∗^*p* < 0.05, ^∗∗^*p* < 0.01, ^∗∗∗^*p* < 0.001 vs. NC + shNC group; ^∧∧^*p* < 0.01, ^∧∧∧^*p* < 0.001 vs. NMT1+shNC group; ^#^*p* < 0.05, ^##^*p* < 0.01 vs. NC + shSPI1 group; ^+^*p* < 0.05, ^++^*p* < 0.01, ^+++^*p* < 0.001 vs. shNMT1+NC group; ^△^*p* < 0.05, ^△△△^*p* < 0.001 vs. shNC + SPI1 group. All experiments were repeated independently at least three times. Data were performed as the means ± standard deviation. NMT1, N-myristoyltransferase 1; GC, gastric cancer; qRT-PCR, quantitative reverse transcription-polymerase chain reaction; GAPDH, glyceraldehyde-3-phosphate dehydrogenase; shNMT1, short hairpin RNA against NMT1; OD, optical density; NC, negative control; shNC, shRNA negative control.

**Figure 6:**
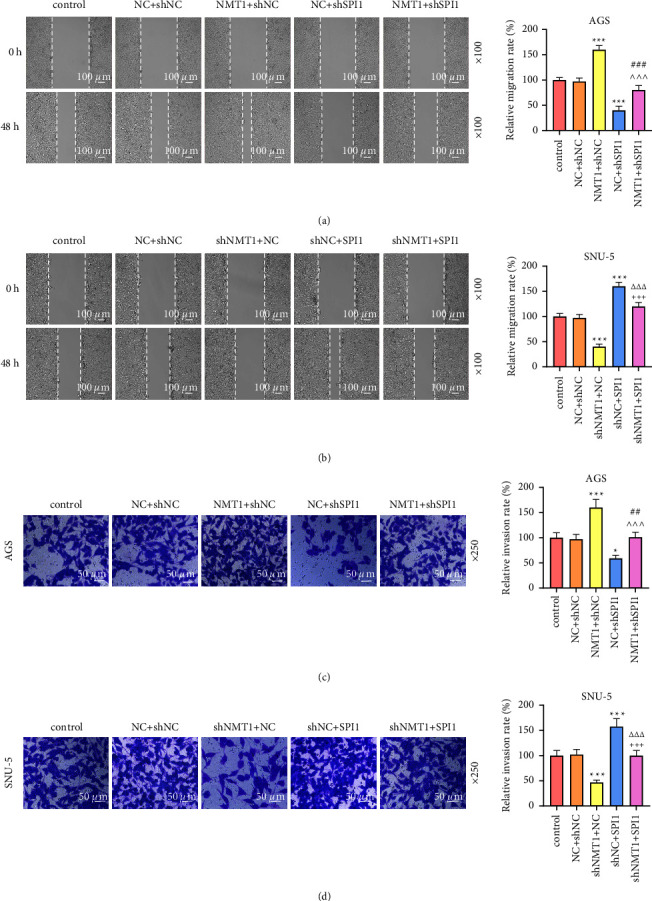
SPI1 regulated NMT1 to mediate GC cell migration and invasion. (a) Migration rate of AGS cells at 48 h was evaluated through wound healing assay after transfection of NMT1 overexpression plasmid and shSPI1. (b) Migration rate of SNU-5 cells at 48 h was evaluated through wound healing assay after transfection of shNMT1 and SPI1 overexpression plasmid. (c) Invasion rate of AGS cells at 48 h was determined by Transwell assay after transfection of NMT1 overexpression plasmid and shSPI1. (d) Invasion rate of SNU-5 cells at 48 h was determined by Transwell assay after transfection of shNMT1 and SPI1 overexpression plasmid. ^∗^*p* < 0.05, ^∗∗∗^*p* < 0.001 vs. NC + shNC group; ^∧∧∧^*p* < 0.001 vs. NMT1+shNC group; ^##^*p* < 0.01, ^###^*p* < 0.001 vs. NC + shSPI1 group; ^+++^*p* < 0.001 vs. shNMT1+NC group; ^△△△^*p* < 0.001 vs. shNC + SPI1 group. All experiments were repeated independently at least three times. Data were performed as the means ± standard deviation. NMT1, N-myristoyltransferase 1; GC, gastric cancer; shNMT1, short hairpin RNA against NMT1; NC, negative control; shNC, shRNA negative control.

**Figure 7:**
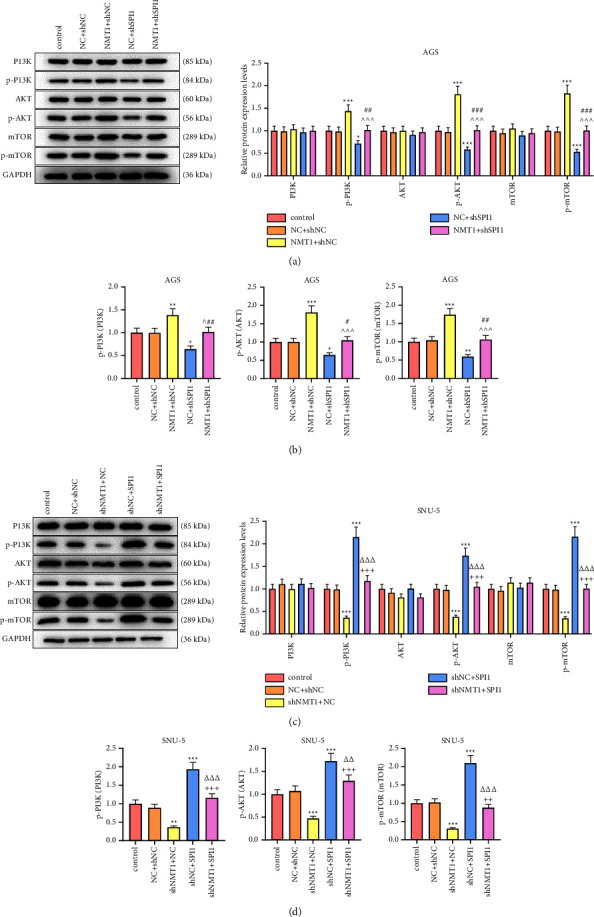
SPI1 regulated NMT1 via PI3K/AKT/mTOR pathway in GC cells. (a) The protein levels of PI3K, p-PI3K, AKT, p-AKT, mTOR, and p-mTOR in AGS cells were tested by western blot after transfection of NMT1 overexpression plasmid and shSPI1. GAPDH was the loading control. (b) The levels of p-PI3K/PI3K, p-AKT/AKT and p-mTOR/mTOR in AGS cells were tested by western blot after transfection of NMT1 overexpression plasmid and shSPI1. GAPDH was the loading control. (c) The protein levels of PI3K, p-PI3K, AKT, p-AKT, mTOR, and p-mTOR in SNU-5 cells were tested by western blot after transfection of shNMT1 and SPI1 overexpression plasmid. GAPDH was the loading control. (d) The levels of p-PI3K/PI3K, p-AKT/AKT and p-mTOR/mTOR in SNU-5 cells were tested by western blot after transfection of shNMT1 and SPI1 overexpression plasmid. GAPDH was the loading control. ^∗^*p* < 0.05, ^∗∗^*p* < 0.01, ^∗∗∗^*p* < 0.001 vs. NC + shNC group; ^∧^*p* < 0.05, ^∧∧∧^*p* < 0.001 vs. NMT1+shNC group; ^#^*p* < 0.05, ^##^*p* < 0.01, ^###^*p* < 0.001 vs. NC + shSPI1 group; ^++^*p* < 0.01, ^+++^*p* < 0.001 vs. shNMT1+NC group; ^△△^*p* < 0.01, ^△△△^*p* < 0.001 vs. shNC + SPI1 group. All experiments were repeated independently at least three times. Data were performed as the means ± standard deviation. NMT1, N-myristoyltransferase 1; GC, gastric cancer; p-, phosphorylation; GAPDH, glyceraldehyde-3-phosphate dehydrogenase; shNMT1, short hairpin RNA against NMT1; NC, negative control; shNC, shRNA negative control.

To verify whether SPI1 participated in the functions of NMT1 on GC, AGS cells were transfected with NMT1 overexpression plasmid and shSPI1 while shNMT1 and SPI1 overexpression plasmid was transfected into SNU-5 cells. As a result, SPI1 overexpression plasmid upregulated SPI1 and NMT1 expression, whereas SPI1 knockdown had the opposite effects (Figures 5(a)–5(d); *p* < 0.01); NMT1 overexpression plasmid increased NMT1 level and shNMT1 restrained NMT1 level (Figures 5(a)–5(d); *p* < 0.01), but both of NMT1 overexpression plasmid and shNMT1 had no influence on SPI1 expression. Contrasted with the NC + shNC group, the NMT1+shNC group elevated the OD value of AGS cells while NC + shSPI1 group decreased that, with the OD value of AGS cells in the NMT1+shSPI1 group was higher than the NC + shSPI1 group and lower than NMT1+shNC group (Figure 5(e); *P* < 0.05). Conversely, SNU-5 cells exhibited a lower OD value in the shNMT1+NC group and a higher OD value in shNC + SPI1 group relative to the NC + shNC group, with the OD value of SNU-5 cells in the shNMT1+SPI1 group was lower than that in the shNC + SPI1 group and higher than that in the shNMT1+NC group (Figure 5(f); *p* < 0.05). The similar consequences were also acquired in the detection of migration and invasion in AGS and SNU-5 cells (Figures 6(a)–6(d); *p* < 0.05). Moreover, with no notable difference observed in PI3K, AKT, and mTOR levels among groups, the NMT1+shNC group increased p-PI3K, p-AKT, and p-mTOR levels of AGS cells and NC + shSPI1 group decreased them in comparison with NC + shNC group, and p-PI3K, p-AKT, and p-mTOR levels of AGS cells in the NMT1+shSPI1 group were higher than those in the NC + shSPI1 group and lower than those in the NMT1+shNC group, leading to a similar results of p-PI3K/PI3K, p-AKT/AKT, and p-mTOR/mTOR (Figures 7(a) and 7(b); *p* < 0.05), whereas the contrary consequences were performed in the SNU-5 cells transfected with shNMT1 and SPI1 overexpression plasmid (Figures 7(c) and 7(d); *p* < 0.001).

## 4. Discussion

NMT1 has been reported to be associated with many cancers [14, 15], including breast cancer, bladder cancer, nonsmall-cell lung cancer, and so on [17, 25, 26]. In this study, the effect of NMT1 in the GC was explored, and we found that NMT1 was significantly upregulated in GC. It is similar to the expression of NMT1 in certain human malignancies such as colorectal cancer, prostate cancer, and breast cancer [15–18]. Moreover, the NMT1 expression level was positively correlated with the poor survival of GC patients. Thus, it could be seen that NMT1 might become a potential biomarker for GC diagnosis and prognosis. A former work has demonstrated that the knockdown of NMT1 could suppress the initiation, growth, and metastasis of breast cancer [17]. During our experiments, overexpression of NMT1 promoted GC cell viability, migration, and invasion while NMT1 knockdown repressed these cell behaviors, consistent with the functions of NOX4, MFN2, as well as Rac1 on GC [27–29], which determines the oncogenic role of NMT1 in GC.

To further gain insight into the regulation of NMT1 in GC, we predicted the transcription factors that may target NMT1 and found that there are two transcription factors in the stomach, KLF5 and SPI1. Current studies on KLF5 in gastric cancer have been extensive [30, 31], but the role of SPI1 is still unknown. SPI1, also known as the transcription factor PU. 1, belongs to the ETS transcription factor family [32]. SPI1 has been reported to be related to the progression of glioma, cervical cancer, breast cancer, and so on [33–35]. SPI1 plays a critical role in regulating the signal communication of the immune system and determining the prognosis of GC patients [36]. In this study, the results showed that SPI1 could bind to NMT1, and SPI1 expression positively affected NMT1 expression in GC cells while NMT1 had no impact on the level of SPI1, which confirmed that the binding relationship between SPI1 and NMT1 in GC cells.

As an oncogenic factor that mediates cell carcinogenesis, SPI1 plays a crucial role in the occurrence and deterioration of cancer [37, 38]. When the SPI1 proto-oncogene is overactivated, a large number of transcription factors SPI1 will be expressed. These SPI1 bind to the promoters of proliferation-related genes, resulting in the overexpression of proliferation proteins, followed by abnormal and uncontrollable activation of cell proliferation mechanisms, and eventually lead to the malignant proliferation of cells [37, 38]. SPI1-induced upregulation of lncRNA SNHG6 promotes nonsmall-cell lung cancer via miR-485-3p/VPS45 axis [39]. Similar to previous studies, our results displayed that the upregulation of SPI1 facilitated cell proliferative, migration, and invasion abilities in GC, whereas the downregulated SPI1 had the opposite effects, with NMT1 partly reversed the effects of SPI1 on the GC cells. These results indicated that SPI1 enhanced the malignant phenotype of GC cells by upregulating NMT1.

The PI3K/AKT/mTOR pathway is considered as a significant immune pathway and has been found to be commonly activated in human cancer [40], such as gastric, prostate, liver, breast, and colorectal cancer, thereby inhibition of this pathway becoming a potential candidate of molecular targeted therapy for malignancies [11, 41–43]. In this study, we found that NMT1 activated the PI3K/AKT/mTOR pathway in GC cells and silencing NMT1 inhibited the PI3K/AKT/mTOR pathway. NMT1 is necessary for lysosomal degradation and mTORC1 activation in cancer cells, and compounds targeting NMT1 may have therapeutic benefit in cancer by preventing mTORC1 activation and simultaneously blocking lysosomal degradation, leading to cancer cell death [44]. Thus, silencing NMT1 may have a therapeutic benefit in GC. In addition, the PI3K/AKT/mTOR pathway regulates cell proliferation, growth, cell size, metabolism, and motility. It has been reported that gene therapy targeting HER-2 promotes tumor cell apoptosis and restrains tumor cell invasion as well as tumor angiogenesis via blocking the PI3K/AKT/mTOR pathway [45]. And another work has revealed that pectolinarigenin inhibits cell cycle progression in GC and induces GC cell autophagy and apoptosis via PI3K/AKT/mTOR pathway [41]. Besides, the PI3K/AKT/mTOR pathway has been proved to be activated in GC and it may have an immunomodulatory potential [46]. In our study, it was found that the NMT1 activated the PI3K/AKT/mTOR pathway in GC cells and partially counteracted the inhibitory functions of silenced SPI1 on the PI3K/AKT/mTOR pathway, implying that the activated signaling pathway was implicated in the regulatory effects of NMT1 on GC. However, the immunomodulatory potential of NMT1 in GC remains obscure and needs more exploration in the future.

## 5. Conclusion

In summary, this research identified NMT1 as a tumor promoter in GC and revealed that SPI1 mediated NMT1 to facilitate GC cell viability, migration, and invasion by activating the PI3K/AKT/mTOR pathway. Our findings implicated the feasibility of NMT1 as a therapeutic target for GC.

## Figures and Tables

**Table 1:** Primer sequences used for quantitative reverse transcription-polymerase chain reaction (qRT-PCR).

Target gene	Primers, 5′-3′
*NMT1*	
(Forward)	CGATTTGATTATTCCCCGGAGTT
(Reverse)	GACTTGAGACCACTCGAACCC

*SPI1*	
(Forward)	AAAATCAGGAACTTGTGCTGGC
(Reverse)	TTGCACGCCTGTAACATCCA

*GAPDH*	
(Forward)	CTGGGCTACACTGAGCACC
(Reverse)	AAGTGGTCGTTGAGGGCAATG

**Table 2:** Primary antibodies in western blot.

Name	Item number	Molecular weight (kDa)	Dilution	Host	Manufacturer
NMT1	ab186123	57	1/2000	Rabbit	Abcam, UK
PI3K	#4292	85	1/1000	Rabbit	CST, USA
p-PI3K	ab278545	84	1/2000	Rabbit	Abcam, UK
AKT	ab8805	60	1/500	Rabbit	Abcam, UK
p-AKT	ab38449	56	1/1000	Rabbit	Abcam, UK
mTOR	ab134903	289	1/10000	Rabbit	Abcam, UK
p-mTOR	ab109268	289	1/10000	Rabbit	Abcam, UK
GAPDH	ab8245	36	1/1000	Mouse	Abcam, UK

## Data Availability

The analyzed data sets generated during the study are available from the corresponding author on reasonable request.
